# NOX5-L can stimulate proliferation and apoptosis depending on its levels and cellular context, determining cancer cell susceptibility to cisplatin

**DOI:** 10.18632/oncotarget.5743

**Published:** 2015-10-15

**Authors:** So Hee Dho, Ji Young Kim, Eun-Soo Kwon, Jae Cheong Lim, Sung Sup Park, Ki-Sun Kwon

**Affiliations:** ^1^ Aging Research Institute, Korea Research Institute of Bioscience and Biotechnology, Daejeon 305–806, Republic of Korea; ^2^ Radioisotope Research Division, Department of Research Reactor Utilization, Korea Atomic Energy Research Institute, Daejeon 305–353, Republic of Korea; ^3^ Department of Functional Genomics, University of Science and Technology (UST), Daejeon 305–333, Republic of Korea

**Keywords:** c-Abl, CREB, cisplatin, NOX5-L, ROS

## Abstract

The NADPH oxidase, NOX5, is known to stimulate cell proliferation in some cancers by generating reactive oxygen species (ROS). We show here that the long form of NOX5 (NOX5-L) also promotes cell death, and thus determines the balance of proliferation and death, in skin, breast and lung cancer cells. Moderate expression of NOX5-L induced cell proliferation accompanied by AKT and ERK phosphorylation, whereas an increase in NOX5-L above a certain threshold promoted cancer cell death accompanied by caspase-3 activation. Notably, cisplatin treatment increased NOX5-L levels through CREB activation and enhanced NOX5-L activity through augmentation of Ca^2+^ release and c-Abl expression, ultimately triggering ROS-mediated cancer cell death—a distinct pathway absent in normal cells. These results indicate that NOX5-L determines cellular responses in a concentration- and context-dependent manner.

## INTRODUCTION

Reactive oxygen species (ROS) were once considered detrimental cellular byproducts; however, they are now widely accepted as signaling molecules that can determine whether cells proliferate or die [1, 2]. ROS stimulate this proliferation switch through several mechanisms [3], including AKT [4, 5] and Ras-ERK (extracellular signal-regulated kinase) pathways [2, 6, 7]. In contrast, it is known that a high level of ROS can trigger cell death by mediating apoptosis through the c-Jun N-terminal kinase and p38 MAPK (mitogen-activated protein kinase) pathways [2, 3]. These pathways are activated by antineoplastic agents that elevate intracellular ROS [8, 9].

Members of the NADPH oxidase (NOX) family are the only enzymes that generate ROS as their main product [10]. They reduce molecular oxygen in an NADPH-dependent fashion to generate superoxide anion [11]. Considering the pleiotropic effects of ROS, NOX activity might contribute to cellular death as well as proliferation. However, until now, most studies have focused on the role of NOX as a mediator of cell proliferation and thus tumorigenesis. For example, the roles of NOX1 in tumorigenesis are well understood in colon [12] and gastric cancers [13], and NOX4 is closely linked to tumorigenesis in pancreatic cancer [14] and melanoma [15].

Compared with the contributions of other NOX members, the function and regulation of NOX5 in tumorigenesis are largely unknown, partly because of limitations in experimental models, as rodents lack NOX5 [16, 17]. Nonetheless, there is a growing consensus that the level of NOX5 is an important factor in certain cancers, such as prostate cancer [18], esophageal adenocarcinoma [19], and melanomas [20].

There are two forms of NOX5 differentiated by the presence or absence of Ca^2+^-binding EF-hands on the *N*-terminus: the long form, NOX5-L, and the short form, NOX5-S [21]. Notably, most NOX5 studies have focused on NOX5-S as a mediator of cancer cell proliferation [19, 21, 22].

In this study, we found that NOX5-L operates as a central switch in the context of tumorigenesis, such that NOX5-L–dependent production of moderate levels of ROS triggers cell proliferation, whereas high levels of NOX5-L promote cancer cell death.

## RESULTS

### An increase in NOX5-L above a certain threshold promotes cancer cell death

**Figure 1:**
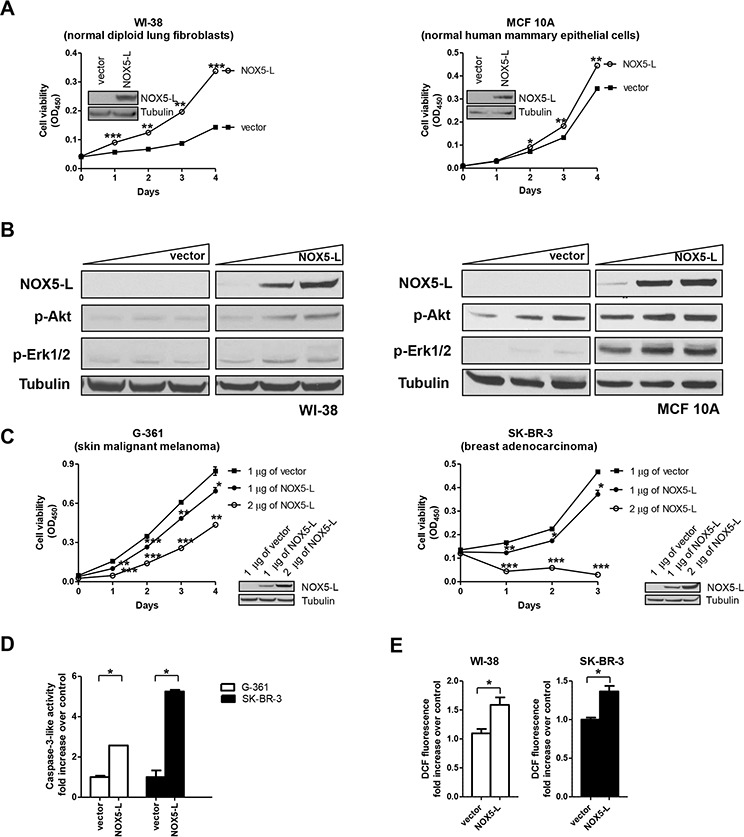
An increase in NOX5-L above a certain threshold promotes cancer cell death **A.** Cell viability assays of WI-38 and MCF10A cells expressing control vector or NOX5-L. *Insets*: Expression of NOX5-L was confirmed by immunoblotting (*n* = 3; *P < 0.05, **P < 0.01, ***P < 0.001 vs. vector; Student's *t* test). **B.** Immunoblots of NOX5-L, p-AKT, p-ERK1/2, and tubulin from WI-38 and MCF10A cells expressing control vector or NOX5-L. **C.** Cell viability assays of G-361 and SK-BR-3 cells expressing control vector or NOX5-L. *Insets*: Expression of NOX5-L was confirmed by immunoblotting (*n* = 3; *P < 0.05, **P < 0.01, ***P < 0.001 vs. vector; Student's *t* test). **D.** Assays of caspase-3-like activity in G-361 and SK-BR-3 cells expressing control vector or NOX5-L (*n* = 2). **E.** Measurement of ROS by dichlorofluorescein (DCF) oxidation. ROS production was measured in WI-38 and SK-BR-3 cells expressing control vector or NOX5-L (*n* = 3).

NOX5 expression has been observed in some cancer cells, but it is rarely detected in normal cells, except those of reproductive and vascular systems [17]. Here, we asked whether NOX5-L overexpression promoted proliferation of normal cells. As expected, NOX5-L overexpression in WI-38 (normal human lung fibroblasts) and MCF10A (normal human mammary epithelial cells) cells induced cell proliferation (Figure 1A) and resulted in production of ROS (Figure 1E). These findings suggest that generation of ROS by NOX5-L promotes cell proliferation.

Next, we sought to identify the mechanism by which NOX5-L induced proliferation in normal cells. To this end, we examined the effect of NOX5-L expression on the activation of the main downstream effectors of tumorigenesis, AKT and ERK1/2, in normal cells. In WI-38 and MCF10A cells, NOX5-L expression led to the phosphorylation of AKT and ERK1/2 in a dose-dependent manner (Figure 1B). We then investigated this effect in cancer cells. Surprisingly, NOX5-L overexpression in G-361 (skin malignant melanoma), SK-BR-3 (breast adenocarcinoma), and HOP-92 (lung carcinoma) cells inhibited cell proliferation (Figure 1C and Supplementary Figure 1). This suggests that NOX5-L promotes cancer cell death when its levels are increased above a certain threshold. We next assessed the cause of cancer cell death and found that increased amounts of NOX5-L promoted apoptosis (Figure 1D). Additionally, NOX5-L expression resulted in production of ROS in cancer cells (Figure 1E). This is also consistent with the fact that high levels of NOX5-L, and therefore high levels of ROS, trigger cell death through apoptosis [2]. Taken together, these results indicate that NOX5-L is a critical regulator of the balance between proliferation and death in cancer cells.

### Cisplatin triggers cell death through enhanced ROS production via NOX5-L upregulation

**Figure 2:**
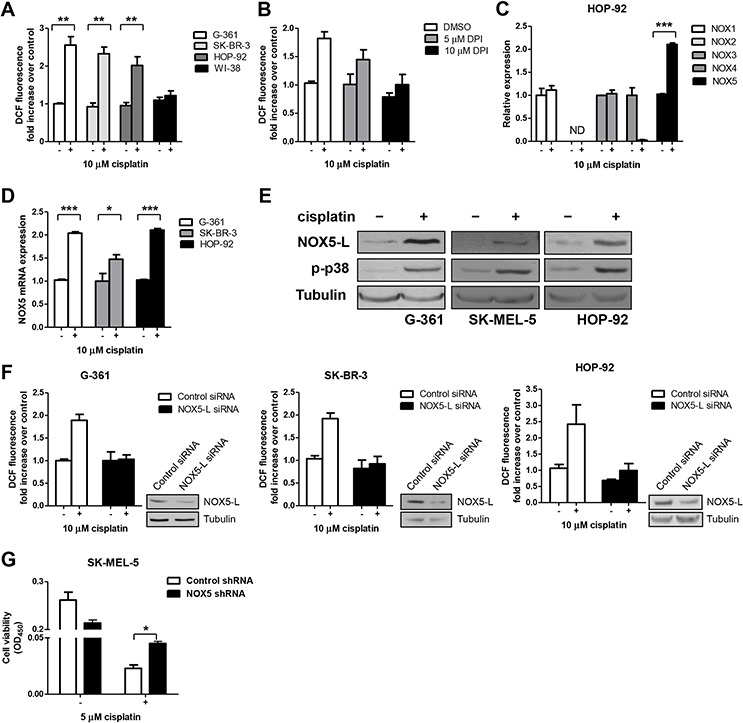
Cisplatin triggers cell death by promoting the production of high ROS levels through NOX5-L upregulation **A.** Measurement of ROS by DCF oxidation in G-361, SK-BR-3, HOP-92, and WI-38 cells. Cells were treated with a clinically relevant concentration of cisplatin (10 μM) [45], and ROS production was measured at 24 h (*n* = 3). **B.** Measurement of ROS by DCF oxidation in HOP-92 cells. Cells were treated with cisplatin and diphenyleneiodonium (DPI) as indicated, and ROS production was measured at 24 h (*n* = 3). **C.** Quantitative RT-PCR of NOX family members in HOP-92 cells. Cells were treated with cisplatin for 24 h (*n* = 3). ND, not detected. **D.** Quantitative RT-PCR of NOX5 in G-361, SK-BR-3, and HOP-92 cells. Cells were treated with cisplatin for 24 h (*n* = 3). **E.** Immunoblots of NOX5-L, p-p38, and tubulin from G-361, SK-MEL-5, and HOP-92 cells treated with cisplatin. p-p38 was used as an indicator of cisplatin treatment because it is a key mediator of stressors such as cisplatin [8]. **F.** Measurement of ROS by DCF oxidation in G-361, SK-BR-3, and HOP-92 cells. Cells expressing control or NOX5-L siRNA were treated with cisplatin, and ROS production was measured at 24 h (*n* = 2). *Insets*: Knockdown of NOX5-L was confirmed by immunoblotting. **G.** Cell viability assays of SK-MEL-5 cells. Cells expressing control or NOX5 shRNA were treated with cisplatin, and cell viability was measured at 72 h (*n* = 2).

Having demonstrated that NOX5-L overexpression triggers cancer cell death (Figure 1), we sought to identify conditions that increase NOX5-L expression. It has been reported that cisplatin induces ROS production [8, 23] and that NOX1 and NOX4 are responsible for cisplatin-induced ROS generation and toxicity in normal auditory [24] and kidney cells [25]. Nevertheless, the effect of NOX on cell death in cisplatin-treated cancer cells is controversial because NOX has also been shown to potentiate cisplatin resistance in glioma [26] and renal cancer cells [27]. Therefore, the exact mechanism by which cisplatin increases ROS and therefore cell death in skin, breast, and lung cancers has not been fully elucidated. We first found that cisplatin treatment increased ROS production in G-361, SK-BR-3, and HOP-92 cells by approximately 2-fold, but did not enhanced ROS generation in WI-38 cells (Figure 2A). These results suggest that cisplatin may kill tumor cells, but spares normal cells because of differential ROS generation.

We next determined whether NOX increases ROS production in response to cisplatin treatment. Diphenyleneiodonium, an inhibitor of NOX, reduced ROS in cisplatin-treated HOP-92 cells in a dose-dependent manner (Figure 2B).

Unlike other NOX family members, NOX5 constitutively produces ROS by itself [28]. This is notable because NOX5 expression levels can be an important determinant of NOX5 activity. To determine whether NOX5 levels are pivotal in cisplatin-induced ROS generation, we examined NOX5 levels after cisplatin treatment. We found that NOX5 levels were increased in cisplatin-treated G-361 and HOP-92 cells (Figure 2C and Supplementary Figure 2A and 2B). A further analysis of these results in G-361, SK-BR-3, and HOP-92 cells using quantitative reverse transcription-polymerase chain reaction (RT-PCR) revealed that NOX5 mRNA levels were increased approximately 2-fold by cisplatin treatment (Figure 2D). Consistent with this result, NOX5-L protein levels were increased in cisplatin-treated cells (Figure 2E).

To confirm the role of NOX5-L in cisplatin-induced ROS generation, we depleted NOX5-L in G-361, SK-BR-3, and HOP-92 cells using small interfering RNA (siRNA) targeting NOX5-L. Cisplatin treatment induced significantly less ROS production in NOX5-L siRNA-transfected cells than in control cells (Figure 2F), indicating that NOX5-L is required for cisplatin-induced ROS production in skin, breast, and lung cancer cells. Finally, shRNA-mediated NOX5-L knockdown experiments showed that cisplatin-induced cell death required NOX5-L (Figure 2G). We note that NOX5-L shRNA did not completely inhibit cisplatin-induced cell death because cisplatin also triggers other signaling pathways and NOX5-L depletion itself induced cell death (unpublished data).

### Cisplatin induces CREB-mediated upregulation of NOX5-L in skin and lung cancer cells

**Figure 3:**
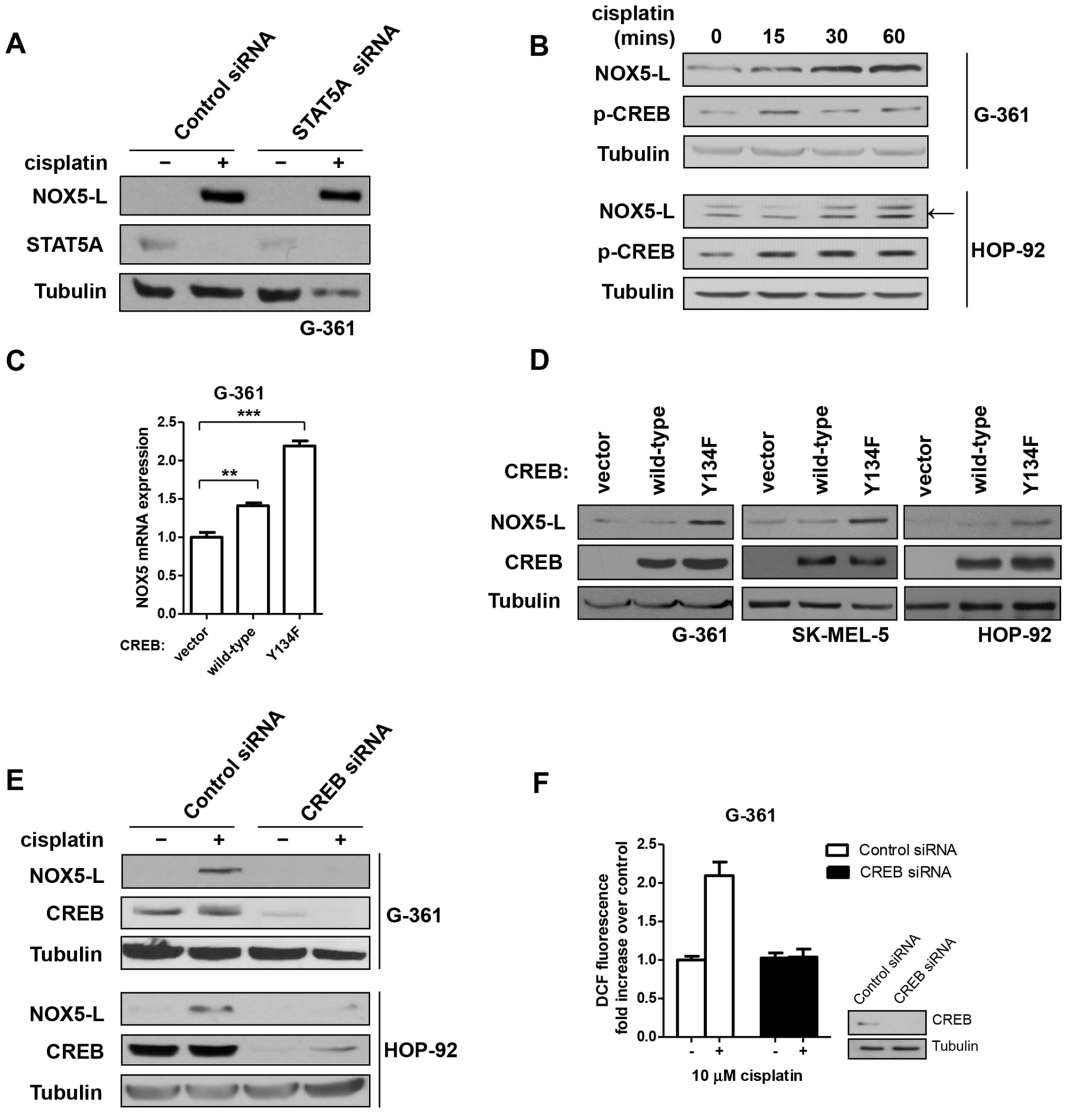
Cisplatin acts through CREB-mediated upregulation of NOX5-L to promote ROS generation in skin and lung cancer cells **A.** Immunoblots of NOX5-L, STAT5A, and tubulin from G-361 cells expressing control or STAT5A siRNA. Cells were treated with cisplatin for 24 h. **B.** Immunoblots of NOX5-L, p-CREB, and tubulin from G-361 and HOP-92 cells. Cells were treated with cisplatin as indicated. Arrow indicates NOX5-L. **C.** Quantitative RT-PCR of NOX5 in G-361 cells expressing control vector, wild-type CREB, or CREB(Y134F) (*n* = 3). **D.** Immunoblots of NOX5-L, CREB, and tubulin from G-361, SK-MEL-5, and HOP-92 cells expressing control vector, wild-type CREB, or CREB(Y134F). **E.** Immunoblots of NOX5-L, CREB, and tubulin from G-361 and HOP-92 cells expressing control or CREB siRNA. Cells were treated with cisplatin for 24 h. **F.** Measurement of ROS by DCF oxidation in G-361 cells. Cells expressing control or CREB siRNA were treated with cisplatin, and ROS production was measured at 24 h (*n* = 3). *Insets*: Expression of CREB was confirmed by immunoblotting.

We next sought to determine which transcription factors are involved in NOX5-L upregulation by cisplatin treatment. Although it has been reported that STAT5 activation contributes to susceptibility to cisplatin [29], we found that STAT5A expression did not alter NOX5-L levels in cisplatin-treated cells (Figure 3A). Notably, cisplatin activates CREB (cAMP response element-binding protein) in ovarian cancer cells [30]. Additionally, CREB mediates acid-induced NOX5-S expression in esophageal adenocarcinoma cells [19], although the promoter region of *NOX5-S* is completely different from that of *NOX5-L*. Therefore, we investigated whether CREB upregulates NOX5-L expression after cisplatin treatment. We found that CREB was activated in response to cisplatin treatment (Figure 3B), and that overexpression of CREB(Y134F), a constitutively active CREB [31], induced the expression of NOX5-L mRNA and protein (Figure 3C and 3D). We then assessed the effect of CREB siRNA on cisplatin-induced NOX5-L upregulation and ROS generation in G-361 and HOP-92 cells. In the presence of cisplatin, G-361 cells transfected with CREB siRNA produced less NOX-5L (Figure 3E) and ROS (Figure 3F) than control siRNA-transfected cells. These findings suggest that CREB upregulates NOX5-L after cisplatin treatment in skin and lung cancer cells.

Interestingly, the endogenous level of NOX5-L was also decreased by CREB knockdown in G-361 and HOP-92 cells (Supplementary Figure 3). Thus, we thought it possible that CREB might also upregulate NOX5-L in the absence of cisplatin in skin and lung cancers; if so, this would suggest that CREB is required for tumorigenesis as well as cisplatin-induced cell death. However, immunohistochemistry showed that only 8% of lung cancer tissues (4 of 50) were positive for phosphorylated (activated) CREB (p-CREB). In addition, even though more skin cancer tissues were p-CREB positive, there was no correlation between NOX5 and activated CREB. Therefore, activated CREB is a critical regulator of NOX5-L transcription in cisplatin-treated lung and skin cancer cells, but not tumorigenesis in these cancers.

### Cisplatin triggers cell death by increasing NOX5-L activity through augmentation of Ca^2+^ release and c-Abl expression

NOX5 can be activated by several factors, including Ca^2+^ [32] and c-Abl [33]. Thus, we assessed whether cisplatin treatment could stimulate NOX5-L activity through these factors.

**Figure 4:**
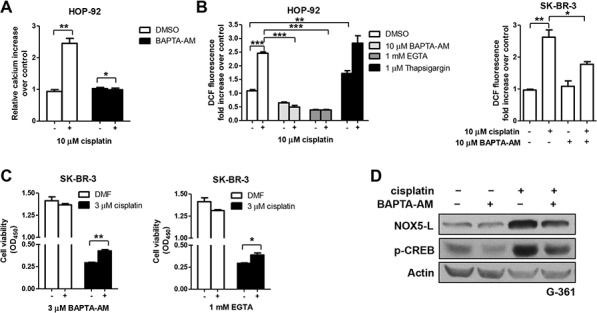
Cisplatin triggers cell death by increasing NOX5-L activity through Ca2+ release **A.** Analysis of Ca^2+^ changes in cisplatin-treated HOP-92 cells by measuring Fluo-4-AM fluorescence (*n* = 3). **B.** Measurement of ROS by DCF oxidation in HOP-92 and SK-BR-3 cells. Cells were treated with DMSO, BAPTA-AM, EGTA, or thapsigargin in the presence or absence of cisplatin (*n* = 3). **C.** Cell viability assays of SK-BR-3 cells. Cells were treated with DMSO, BAPTA-AM, or EGTA in the presence or absence of cisplatin (*n* = 3). **D.** Immunoblots of NOX5-L, p-CREB, and actin from G-361 cells. Cells were treated with DMSO or BAPTA-AM in the presence or absence of cisplatin.

We first found that cisplatin treatment increased intracellular Ca^2+^ levels (Figure 4A). Next, we assessed whether ROS production, indicative of NOX5-L activation, is increased by cisplatin-induced Ca^2+^ elevation. Chelation of intracellular Ca^2+^ with BAPTA-AM and EGTA abolished ROS production in cisplatin-treated HOP-92 and SK-BR-3 cells (Figure 4B). Additionally, co-treatment with cisplatin and thapsigargin, the latter of which induces Ca^2+^ release, activated NOX5-L further, as demonstrated by ROS generation in HOP-92 cells (Figure 4B). Consistently, NOX5-L activation by cisplatin-induced Ca^2+^ release was correlated with cancer cell death. Ca^2+^ depletion reduced cisplatin-induced cell death (Figure 4C); however, it did not entirely block the cell death, likely because other mediators of cisplatin stimulation might still operate even in the absence of Ca^2+^. We then considered the possibility that cisplatin-induced Ca^2+^ release increases NOX5-L levels as well as NOX5-L activity. We found that Ca^2+^ depletion by BAPTA-AM suppressed cisplatin-induced increases in NOX5-L levels in association with decreased CREB activation (Figure 4D), indicating that the increase in intracellular Ca^2+^ concentration caused by cisplatin treatment acts through CREB activation to upregulate NOX5-L. Therefore, cisplatin-induced activation of NOX5-L is due, at least in part, to Ca^2+^-dependent induction of NOX5-L expression.

**Figure 5:**
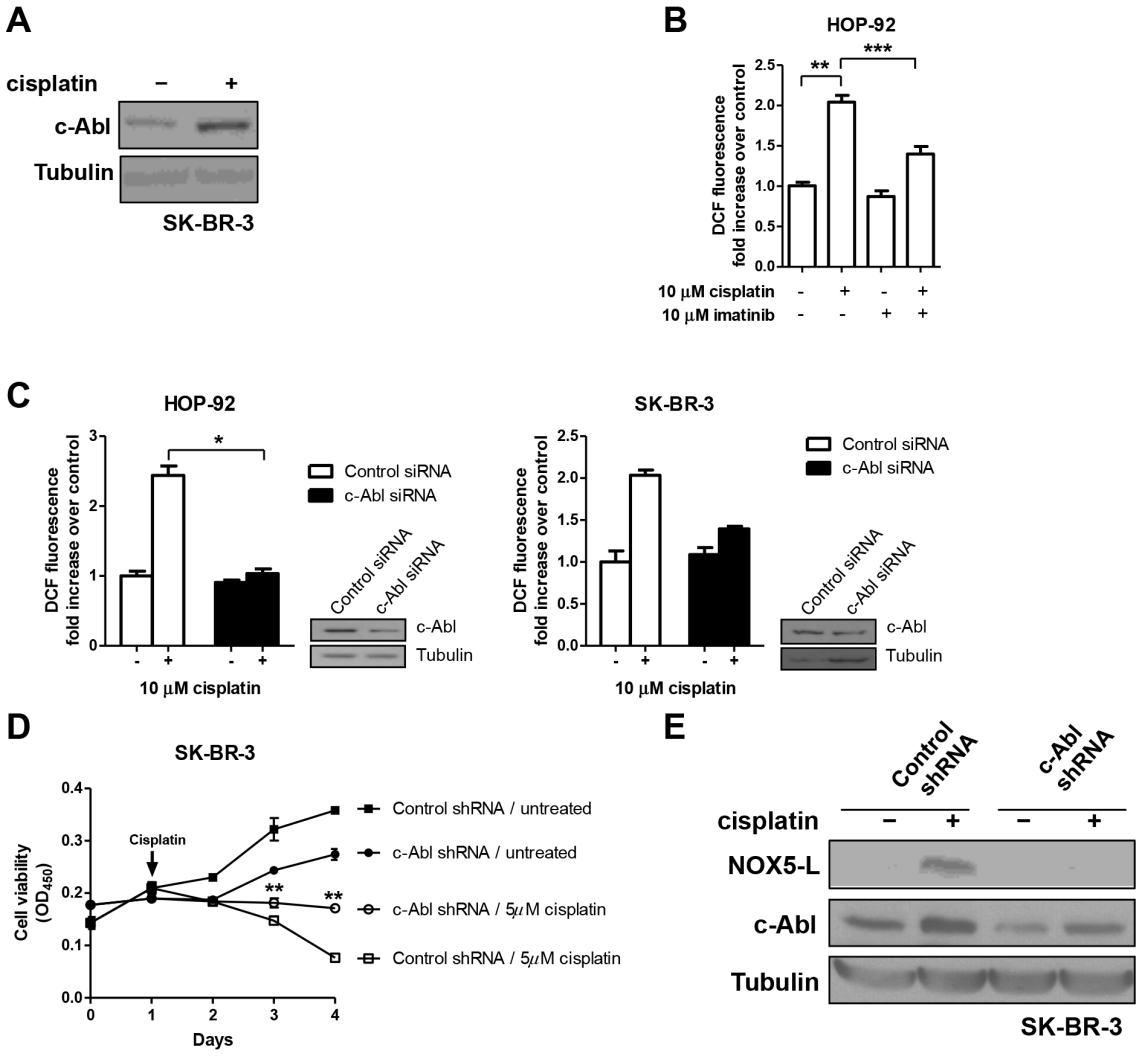
Cisplatin triggers cell death by increasing NOX5-L activity through c-Abl augmentation **A.** Immunoblots of c-Abl and tubulin from SK-BR-3 cells. Cells were treated with cisplatin for 24 h. **B.** Measurement of ROS by DCF oxidation in HOP-92 cells. Cells were treated with DMSO or imatinib in the presence or absence of cisplatin (*n* = 4). **C.** Measurement of ROS by DCF oxidation in HOP-92 and SK-BR-3 cells. Cells expressing control or c-Abl siRNA were treated with cisplatin (*n* = 3). *Insets*: Expression of c-Abl was confirmed by immunoblotting. **D.** Cell viability assays of SK-BR-3 cells expressing control or c-Abl shRNA (*n* = 3; **P < 0.01 vs. cisplatin-treated control shRNA; Student's *t* test). **E.** Immunoblots of NOX5-L, c-Abl, and tubulin from SK-BR-3 cells. Cells were infected with control or c-Abl shRNA in the presence or absence of cisplatin.

We next found that cisplatin treatment increased c-Abl levels (Figure 5A), suggesting that c-Abl might activate NOX5-L in cisplatin-treated cells. Interestingly, imatinib, a c-Abl inhibitor, protects against cisplatin-induced ovarian follicle loss [34] and suppresses cisplatin-induced cell death in breast cancer cells [35]. Nevertheless, the effect of imatinib on cisplatin-treated cancer cells is controversial because imatinib has also been shown to potentiate cisplatin sensitivity [36]. To clarify the effect of imatinib on cisplatin-treated cells, we treated HOP-92 cells with both agents. We found that cisplatin-induced ROS generation was decreased by imatinib (Figure 5B) and siRNA specific for c-Abl (Figure 5C). We also asked whether cisplatin-induced cell death requires c-Abl. Under c-Abl–depleted conditions, cisplatin caused less cell death compared with control conditions (Figure 5D), suggesting that c-Abl stimulates NOX5-L-mediated ROS production, and ultimately promotes cell death in cisplatin-treated cells.

Finally, we considered the possibility that, as was shown for Ca^2+^, c-Abl was required for upregulation of NOX5-L levels and activity by cisplatin treatment. We found that c-Abl knockdown decreased cisplatin-induced expression of NOX5-L (Figure 5E). Thus, NOX5-L activation after cisplatin treatment is at least partially a consequence of a c-Abl–dependent increase in NOX5-L levels.

## DISCUSSION

**Figure 6:**
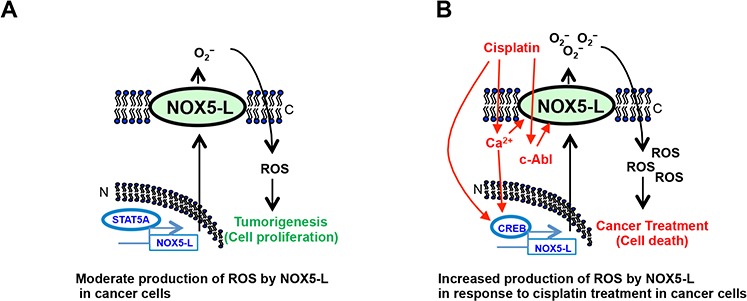
Schematic showing that NOX5-L is a critical regulator of the balance between proliferation and death in cancer cells Red, black, and blue represent cell death, ROS generation, and NOX5-L transcription, respectively. C, cytoplasmic membrane; N, nuclear membrane.

NOX isoforms have hitherto received attention as regulators of cancer cell proliferation. Here, we focused on a binary role of NOX5-L in both proliferation and death of cancer cells that has not been previously reported. We show that proliferation and death are promoted by two different transcriptional regulators of NOX5-L in cancer cells: STAT5A (unpublished data) and CREB, respectively. In the case of cancer cell death, Ca^2+^ and c-Abl are critical mediators of NOX5-L activation (Figure 6). Cisplatin increased both the level and activity of NOX5-L in cancer cells, thereby inhibiting cell proliferation.

Cisplatin exerts its tumoricidal activity through DNA-damage–induced cell signaling [37]; however, the selectivity towards cancer cells cannot be explained by DNA damage because cisplatin causes the formation of DNA adducts in both normal and cancer cells [38]. One possible explanation for this selectivity that has been advanced is based on the observation that Bcl-x_L_ undergoes deamidation only in susceptible cancer cells [38, 39]. In the current study, we provide another basis for the selectivity of cisplatin towards cancer cells: ROS production generated by NOX5-L. In response to cisplatin, ROS were produced only in cancer cells and not in normal cells (Figure 2A). Therefore, NOX5-L activity, and thus ROS generation, might be used as a marker to predict sensitivity to cisplatin.

Even though the initial response to cisplatin treatment is usually outstanding, resistance becomes a major challenge. The typical approach for overcoming such resistance is administration of combinatorial therapy [37]. Our findings suggest that the activity of NOX5-L may be pivotal for decisions regarding combinatorial anticancer therapy with cisplatin for a number of reasons. (i) Cisplatin and antineoplastic agents that increase intracellular Ca^2+^ may have additive effects on cancer treatment. Because cisplatin-induced intracellular Ca^2+^ release activates NOX5-L, additional Ca^2+^ induced by other antineoplastic agents would further activate NOX5-L and thus enhance ROS-mediated cell death. (ii) Anti-hypertension medications that antagonize Ca^2+^, such as nifedipine and amlodipine, may decrease cisplatin efficacy. Ca^2+^ antagonists can counteract the effect of cisplatin-induced Ca^2+^ release, thus inhibiting NOX5-L activation. (iii) Imatinib may inhibit cisplatin efficacy. Although cisplatin induced an increase in c-Abl, which activates NOX5-L, imatinib inhibited c-Abl and therefore suppressed NOX5-L.

Although we have demonstrated that CREB, Ca^2+^, and c-Abl are critical regulators of NOX5-L in cisplatin-induced cell death, we do not exclude the possibility that other mechanisms, including ubiquitination [40] and phosphorylation [41], or other regulators also affect NOX5-L levels and activity.

## MATERIALS AND METHODS

### Cell culture

SK-MEL-5 (skin malignant melanoma; ATCC HTB-70) cells were maintained in MEMα with 10% fetal bovine serum (FBS). SK-BR-3 (breast adenocarcinoma; ATCC HTB-30) and G-361 (skin malignant melanoma; ATCC CRL-1424) cells were maintained in McCoy's 5A with 10% FBS. HOP-92 (lung carcinoma) [42] cells were maintained in RPMI-1640 with 10% FBS. WI-38 (normal human lung fibroblasts; ATCC CCL-75) cells were maintained in DMEM with 10% FBS. MCF10A (normal human mammary epithelial cells; ATCC CRL-10317) cells were maintained in DMEM/F12 with 10 μg/mL insulin, 0.5 mg/mL hydrocortisone, 20 ng/mL EGF (epidermal growth factor), 100 ng/mL cholera toxin, and 10% FBS.

### Plasmids, transfection, and infection

pcDNA3.1-NOX5-L was a generous gift from Dr. Fulton [41]. pCG-CREB was a generous gift from Dr. Suh-Kim [43]. Codon 134 of the CREB gene was changed from TAC to TTC to generate CREB(Y134F) using QuikChange (200518; Stratagene).

For NOX5 knockdown, we used five lentiviral pLKO.1 plasmids containing shRNAs against NOX5 sequences (TRCN0000046098, TRCN0000046099, TRCN0000046100, TRCN0000046101, and TRCN0000046102; Sigma) and an siRNA against NOX5 EF-hand sequences (1104585; Bioneer). For CREB knockdown, we used five lentiviral pLKO.1 plasmids containing shRNAs against CREB sequences (TRCN0000226466, TRCN0000226467, TRCN0000226468, TRCN0000226469, and TRCN0000007308; Sigma) and two siRNAs against CREB sequences (1035590 and 1035585; Bioneer). For c-Abl knockdown, we used five lentiviral pLKO.1 plasmids containing shRNAs against c-Abl sequences (TRCN0000039898, TRCN0000039899, TRCN0000039900, TRCN0000039901, and TRCN0000039902; Sigma) and two siRNAs against c-Abl sequences (100523 and 100524; Bioneer).

Cells were transfected using Nucleofector (Amaxa). Standard lentiviral techniques were used for shRNA infection. Cell viability was quantified with a Cell Counting kit-8 (CK04–11; Dojindo Molecular Technologies) by measuring absorbance at 450 nm using a microplate reader. Caspase-3-like activity was measured using a Caspase-3 Activity Assay Kit (5723; Cell Signaling).

Cells were treated with 10 μM cisplatin for 24 h unless otherwise indicated. One hour before cisplatin treatment, cells were pretreated with 5 or 10 μM DPI, 10 μM BAPTA-AM, 1 mM EGTA, 1 μM thapsigargin, or 10 mM imatinib, as indicated in the text.

### Immunoblotting and immunohistochemistry

The following antibodies were used for immunoblotting: anti-NOX5 (a generous gift from Dr. Nauseef); anti-phospho-AKT (4060), anti-phospho-ERK1/2 (4370), anti-phospho-CREB (9198), anti-CREB (9197), and anti-phospho-p38 (4511) from Cell Signaling; anti-NOX5 (ab178731), anti-c-Abl (ab15130), anti-Duox1 (ab178534), and anti-Duox2 (ab97266) from Abcam; and anti-β-tubulin (sc-5274) and anti-β-actin (sc-1616) from Santa Cruz Biotechnology. Immunoblotting was performed as previously described [44].

### Measurement of ROS levels

ROS generation was determined using 2′,7′-dichlorodihydrofluorescein diacetate (H_2_DCFDA; Molecular Probes). Cells were incubated with 20 μM H_2_DCFDA for 45 min at 37°C, trypsinized, washed, resuspended in phosphate-buffered saline (PBS), and immediately analyzed using a Victor X3 Multilabel Plate Reader (Perkin Elmer; excitation 485 nm, emission 535 nm).

### Quantitative and conventional RT-PCR

Total RNA was isolated using QIAzol reagent (79306; Qiagen). cDNA was synthesized from 2 μg of total RNA using a DiaStar RT Kit (DR-23-R10K; SolGent). Real-time quantitative PCR was performed using iQ SYBR Green Supermix (170–8882AP; Bio-Rad) and specific primers (Supplementary Table 1) on a Bio-Rad CFX96 system. The following thermocycling conditions were used: 95°C for 3 min, followed by 40 cycles of 95°C for 20 s, 60°C for 20 s, and 72°C for 20 s. The threshold cycle (Ct) was defined as the fractional cycle number at which the fluorescence exceeded a fixed threshold. The GAPDH (glyceraldehyde-3-phosphate dehydrogenase) housekeeping gene served as an endogenous control for normalization. Quantitative and conventional RT-PCR primer sequences are listed in Supplementary Tables 1 and 2.

### Ca^2+^ imaging

HOP-92 cells were plated on μ-Slide VI^0.4^chambers (Ibidi) 24 h prior to experiments and loaded with the Ca^2+^-binding fluorescent dye Fluo-4-AM dye (5 μM; Molecular Probes) for 30 min at 37°C in physiological salt solution (150 mM NaCl, 4 mM KCl, 2 mM CaCl_2_, 1 mM MgCl_2_, 5 mM glucose, and 5 mM HEPES). The cells were treated with 10 μM cisplatin in the presence or absence of 10 μM BAPTA-AM (Molecular Probes). Ca^2+^ was imaged with an inverted microscope (LSM 510 META and LIVE 5; Carl Zeiss) equipped with a 40× objective (excitation 488 nm, emission 505/530 nm).

## SUPPLEMENTARY FIGURES AND TABLES


