# Supplementary material for: NOX5-L can stimulate proliferation and apoptosis depending on its levels and cellular context, determining cancer cell susceptibility to cisplatin
Source: Oncotarget. 2015 Oct 15;6(36):39235–46. doi: 10.18632/oncotarget.5743 (PMC4770769; doi:10.18632/oncotarget.5743)
Supplement: Supplementary file 1 [file oncotarget-06-39235-s001.pdf]

SUPPLEMENTARY FIGURES AND TABLES

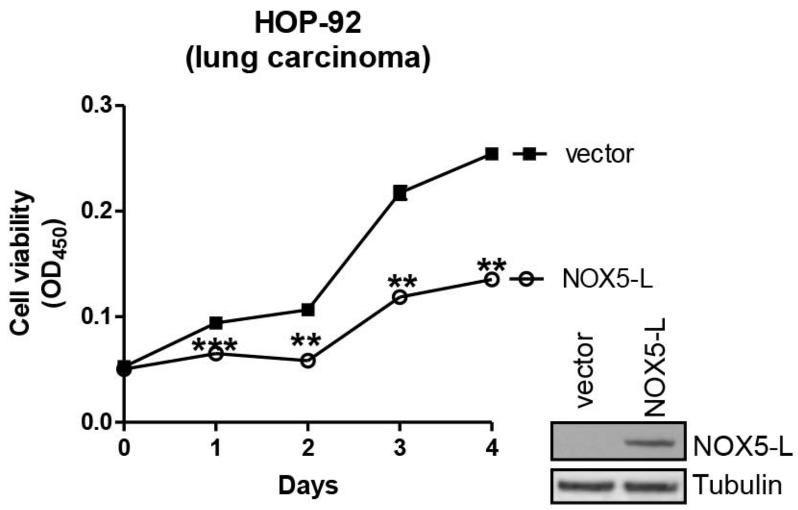

Supplementary Figure S1: NOX5-L expression causes cell death in HOP-92 cells. Cell viability assays of HOP-92 cells expressing control vector or NOX5-L. *Insets*: Expression of NOX5-L was confirmed by immunoblotting ($n = 3$; $^{**}P < 0.01$, $^{***}P < 0.001$ vs. vector; Student's t test).

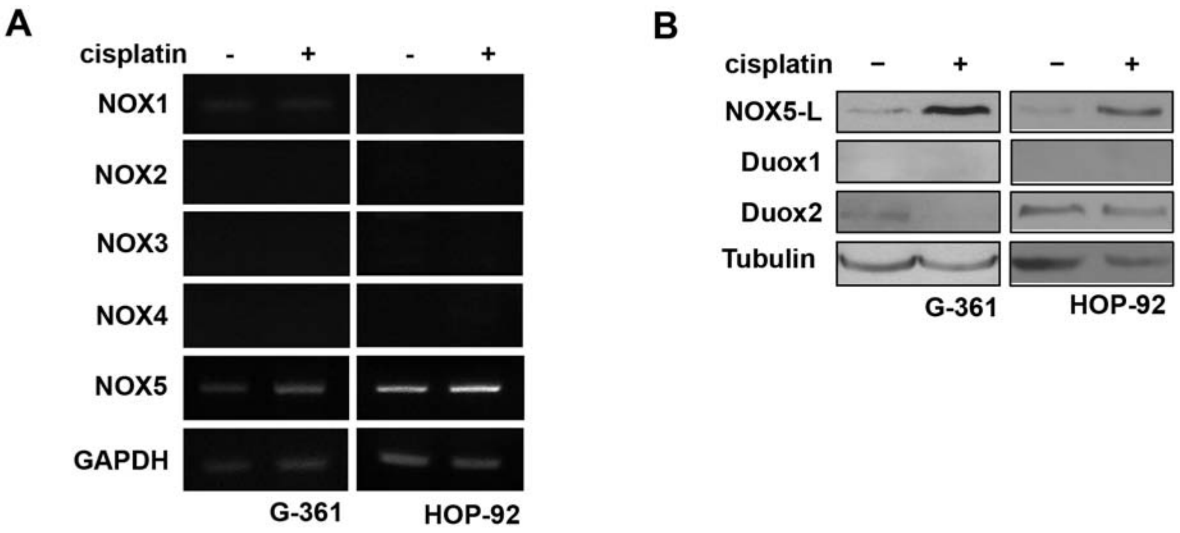

Supplementary Figure S2: Cisplatin triggers the production of high ROS levels through NOX5-L upregulation. mRNA **A.** and protein **B.** expression of NOX family members in G-361 and HOP-92 cells. Cells were treated with cisplatin for 24 h. GAPDH and tubulin were used as loading controls.

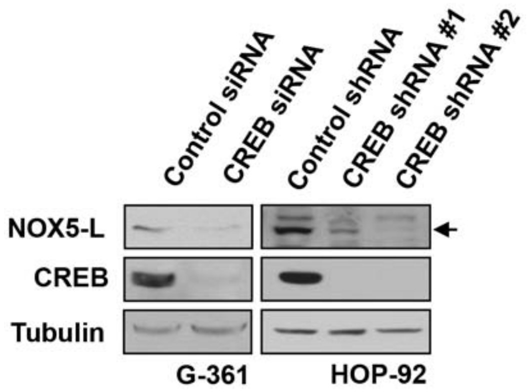

Supplementary Figure S3: CREB knockdown downregulates NOX5-Lin cancer cells. Immunoblots of NOX5-L, CREB, and tubulin from G-361 and HOP-92 cells expressing control, CREB siRNA or CREB shRNA. Arrow indicates NOX5-L.

Supplementary Table S1: Quantitative RT-PCR primer sequences

Target	Forward (5′-3′)	Reverse (5′-3′)
Human NOX1	GGAGCAGGAATTGGGGTCAC	TTGCTGTCCCATCCGGTGAG
Human NOX2	GGAGTTTCAAGATGCGTGGAAACTA	GCCAGACTCAGAGTTGGAGATGCT
Human NOX3	GGATCGGAGTCACTCCCTTCGCTG	ATGAACACCTCTGGGGTCAGCTGA
Human NOX4	GGAGTTTCAAGATGCGTGGAAACTA	GCCAGACTCAGAGTTGGAGATGCT
Human NOX5	CTATTGGACTCACCTGTCCTACC	GGAAAAACAAGATTCCAGGCAC
Human GAPDH	TGTTGCCATCAATGACCCCTT	CTCCACGACGTACTCAGCG

Supplementary Table S2: RT-PCR primer sequences

Target	Forward (5′-3′)	Reverse (5′-3′)
Human NOX1	GCACACCTGTTTAACTTTGACTG	GGACTGGATGGGATTTAGCCA
Human NOX2	CATCACCAAGGTGGTCACTC	TCAACCGCTATCTTAGGTAGTTT
Human NOX3	ACTGCCCTGACAGATGTATT	CTGCTTGAACTCATTGTTCC
Human NOX4	GACTGGACAGAACGATTTCG	CTTGCCAAAACTTGTTATGCA
Human NOX5	TCATCTCCTCATCAAGCGGC	ATGAGCACGGCATGCTCAGA
Human GAPDH	TGTTGCCATCAATGACCCCTT	CTCCACGACGTACTCAGCG
